# The effect of intraperitoneal St. John’s Wort oil (*Hypericum perforatum*) in an experimental peritonitis model

**DOI:** 10.1590/acb416026

**Published:** 2026-10-19

**Authors:** Yunus Kayci, Burak Yavuz, Ahmet Onur Demirel, Ibrahim Çoğal, Zülfi Zahidli, Kivilcim Eren Ateş, Uğur Topal, Ismail Cem Eray, Mustafa Yalçın Kekeç

**Affiliations:** 1 University of Health Sciences – Adana City Training and Research Hospital – Department of Gastroenterological Surgery – Adana – Turkey.; 2 Çukurova University – Faculty of Medicine – Department of General Surgery – Adana – Turkey.; 3 Çukurova University – Faculty of Medicine – Department of Pathology – Adana – Turkey.

**Keywords:** Hypericum, Peritonitis, Sepsis, Cytokines, Disease Models, Animal

## Abstract

**Purpose:**

To evaluate the effects of intraperitoneal *Hypericum perforatum* oil (St. John’s Wort oil, SJWO) on systemic inflammation and peritoneal histopathology in experimental peritonitis.

**Methods:**

Forty-two male Wistar Albino rats were randomized into seven groups (n = 6): control, SJWO, olive oil, peritonitis, peritonitis + saline, peritonitis + SJWO, and peritonitis + olive oil. Peritonitis was induced with freshly prepared autologous fecal suspension, and treatments were administered intraperitoneally at 48 h. On day 5, plasma inflammatory markers, peritoneal thickness, and histopathological scores were assessed blinded.

**Results:**

The greatest weight loss was seen in the peritonitis group (*p* = 0.002). SJWO did not consistently reduce systemic inflammatory markers. Compared with peritonitis + saline, peritonitis + SJWO showed lower parietal fibrosis and vascular proliferation scores (rank-biserial correlation for each outcome, *r*_rb_ = -0.833; 95% confidence interval -1.000 to -0.500), but visceral fibrosis was not reduced.

**Conclusion:**

Intraperitoneal SJWO did not suppress systemic inflammation. The lower early parietal fibrosis and vascular proliferation scores are exploratory because the effect was not observed in visceral tissue, and adhesion formation was not directly assessed.

## Introduction

Sepsis is a major cause of mortality, and its incidence is rising. Its current global incidence is estimated at 18–30 million cases per year. When sepsis is accompanied by shock, mortality rates may reach 45% in developed countries and up to 85% in developing countries^1^. Intra-abdominal sepsis follows an aggressive course and is the second most common cause of sepsis^2^.

Spread of intra-abdominal infections to the peritoneum is termed peritonitis and is classified as primary, secondary, or tertiary. The most frequently form encountered in surgical practice is secondary peritonitis, which develops following loss of gastrointestinal tract integrity; the cornerstones of management are source control, reduction of contamination, and prevention of recurrent infection^3^. Enteric microorganisms reaching the systemic circulation may lead to sepsis, septic shock, multi-organ failure, and death.

In the management of intra-abdominal sepsis, the underlying pathology must be promptly identified; surgical treatment, intravenous antibiotics, fluid resuscitation, and intensive care support should be delivered concurrently^4^. Despite all these advances, peritonitis and intra-abdominal sepsis continue to be associated with high morbidity and mortality. The bulk of research on peritonitis has focused on reducing intra-abdominal contamination and bacterial translocation; to this end, numerous chemicals, antibiotics, herbal products, and peritoneal lavage protocols using normal saline or hypochlorous acid have been investigated^3,4^.

*Hypericum perforatum* (St. John’s Wort) is a medicinal plant reported to have antidepressant, antimicrobial, antiviral, anti-inflammatory, wound-healing, and epithelialization-enhancing properties. Several clinical and experimental studies have described its beneficial effects in tympanic membrane perforations and pressure ulcers^5,6^. However, no data are available on the intraperitoneal use of St. John’s Wort oil (SJWO) in an experimental peritonitis model.

The aim of the present study was to investigate the effects of intraperitoneal SJWO administration on systemic inflammatory markers and peritoneal histopathology in a feces-induced experimental peritonitis model.

## Methods

### Ethical statement

This study was approved by the Local Animal Ethics Committee of Çukurova University at its 8th meeting on 17 September 2021 (decision no. 2) and was conducted at the Çukurova University Health Sciences Experimental Research and Application Center in accordance with the Animal Research: Reporting of In Vivo Experiments (ARRIVE) 2.0 guidelines.

### Animals and housing

Forty-two Wistar Albino rats weighing 240–300 g were used. Throughout the experiment, a 12-h light/12-h dark cycle was maintained; animals were housed six per cage in standard cages at an average ambient temperature of 21°C and were fed standard pellet chow and *ad libitum* filtered tap water. In accordance with the Sex and Gender Equity in Research (SAGER) guidelines, male Wistar Albino rats were used to maintain hormonal homogeneity.

### Experimental peritonitis model

For each rat, fresh feces were collected separately from that same animal at 0.5 mL/kg body weight and suspended in 1 mL of sterile 0.9% sodium chloride. Samples from different rats were not pooled, thereby avoiding exposure to another animal’s distinct fecal microbiota. Each autologous suspension was mechanically agitated until macroscopically homogeneous and was administered immediately after preparation without storage. Following sedation with sevoflurane, 1 mL of the final suspension was injected intraperitoneally through the right lower quadrant using a 16-gauge needle. The abdomen was then gently massaged with two fingers to distribute the inoculum. Procedural standardization was maintained by using the same weight-adjusted fecal amount, diluent volume, mixing procedure, final administered volume, route, and preparation-to-injection interval for every rat.

### St. John’s Wort oil preparation and dose rationale

A commercially available 50-mL olive-oil-based macerate of *H. perforatum* was obtained from Murat Sami (Iskenderun, Hatay, Turkey). Its lot number, plant-to-oil ratio, detailed extraction conditions, quantitative standardization, and hypericin/hyperforin concentrations were not available, and no independent phytochemical analysis was performed. Hizli et al.^7^ administered 2 mL/rat of an intraperitoneal *H. perforatum* preparation in a rat adhesion model. In the present peritonitis model, 1 mL/rat was selected to limit the intraperitoneal fluid load while maintaining direct peritoneal exposure; the same 1-mL volume was used in all saline, SJWO, and olive-oil intervention groups.

### Experimental groups

Rats were assigned to seven groups of six animals each (Table 1):

**Table 1:** Composition of the experimental groups.

Group	Fecal suspension	Normal saline	St. John's Wort oil	Olive oil
Control (n = 6)	−	−	−	−
Peritonitis (n = 6)	+	−	−	−
Peritonitis + saline (n = 6)	+	+	−	−
Peritonitis + SJWO (n = 6)	+	−	+	−
Peritonitis + olive oil (n = 6)	+	−	−	+
SJWO (n = 6)	−	−	+	−
Olive oil (n = 6)	−	−	−	+

+: administration of the agent; −: no administration; SJWO: St. John’s Wort oil. Source: Elaborated by the authors.

- Group 1 (control, n = 6): no intraperitoneal intervention;
- Group 2 (peritonitis, n = 6): intraperitoneal injection of fecal suspension on day 0;
- Group 3 (peritonitis + saline, n = 6): fecal suspension on day 0; 1 cc of normal saline injected intraperitoneally on day 2;
- Group 4 (peritonitis + SJWO, n = 6): fecal suspension on day 0; 1 cc of St. John’s Wort oil injected intraperitoneally on day 2;
- Group 5 (peritonitis + olive oil, n = 6): fecal suspension on day 0; 1 cc of olive oil injected intraperitoneally on day 2;
- Group 6 (SJWO, n = 6): 1 cc of St. John’s Wort oil injected intraperitoneally on day 2;
- Group 7 (olive oil, n = 6): 1 cc of olive oil injected intraperitoneally on day 2.

Because St. John’s Wort oil is obtained as a mixture of *H. perforatum* extract and olive oil, the olive oil (group 7) and peritonitis + olive oil (group 5) groups were included to assess the possible independent effect of the vehicle. All groups were maintained under standard housing and feeding conditions throughout the experiment.

### Sacrifice and sampling

At the end of the fifth day, all rats were weighed, and their final body weights were recorded. Anesthesia was achieved by intraperitoneal injection of ketamine hydrochloride 50 mg/kg and xylazine hydrochloride 5 mg/kg, after which a midline incision was performed for laparotomy and sternotomy (Fig. 1). Blood obtained by intracardiac sampling from the right atrium was collected in citrate tubes and centrifuged at 5,000 rpm for 5 min. The separated plasma was stored in Eppendorf tubes; interleukin (IL)-1, IL-6, tumor necrosis factor (TNF)-α, C-reactive protein (CRP), and procalcitonin levels were measured from these samples.

**Figure 1:**
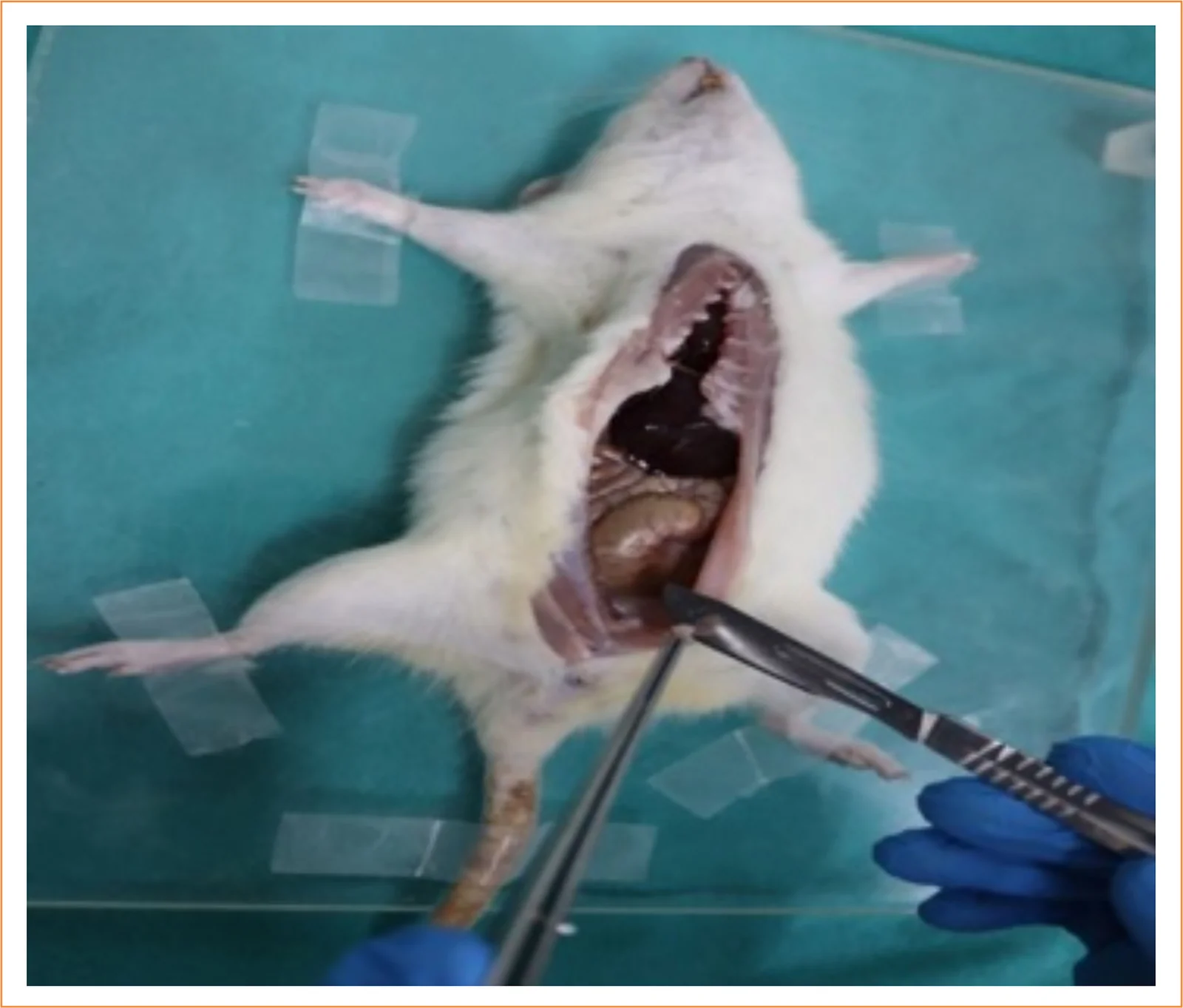
Midline laparotomy and sternotomy performed in a rat at the end of the experimental protocol, prior to intracardiac blood sampling and tissue collection.

Source: Elaborated by the authors.

### Blinded biochemical analysis

Blood samples and separated plasma aliquots were labelled with coded identifiers that did not disclose the rat identity or experimental group before transfer to the biochemistry laboratory. CRP, procalcitonin, IL-1, IL-6, and TNF-α were measured by laboratory personnel who were unaware of sample identity and group allocation. The allocation code was released only after all biochemical measurements had been completed and the results recorded.

### Histopathological evaluation

Parietal peritoneal samples were taken from the parietal peritoneum, and visceral peritoneal samples from the hepatic capsule. Tissue specimens were fixed in 10% sterile formaldehyde solution. Specimens were then cut into 0.5-cm pieces, embedded in paraffin blocks, sectioned at 5 µm, and stained with hematoxylin–eosin. All slides were evaluated by a single expert pathologist blinded to group assignment using light microscopy. Tissue thickness was measured in micrometers; inflammation, fibrosis, and vascular proliferation were semi-quantitatively scored on a 0–3 scale (Table 2).

**Table 2:** Semi-quantitative scoring system for tissue inflammation, fibrosis, and vascular proliferation.

Parameter	Normal	Mild	Moderate	Severe
Inflammation	0	1	2	3
Fibrosis	0	1	2	3
Vascular proliferation	0	1	2	3

0: normal; 1: mild; 2: moderate; 3: severe. Source: Elaborated by the authors.

### Statistical analysis

The original hypothesis tests were performed using IBM Statistical Package for the Social Sciences Statistics version 20.0 (IBM Corp., Armonk, NY, United States of America). Effect-size and bootstrap calculations for this revision were performed using Python version 3.12.13 with SciPy version 1.17.0 and NumPy version 2.3.5. Categorical variables were summarized as number and percentage and numerical variables as mean ± standard deviation, with median (minimum-maximum) reported when appropriate. The Shapiro-Wilk’s test was used to assess normality. For comparisons across seven groups, one-way analysis of variance (ANOVA) was used for variables meeting parametric assumptions and the Kruskal-Wallis’ test for variables that did not. Pairwise comparisons were performed using the Bonferroni post-hoc test or Mann-Whitney’s U test with Bonferroni correction. To complement *p*-values, omnibus effect sizes were calculated as omega-squared (ω^2^) for one-way ANOVA and epsilon-squared (ε^2^) for Kruskal-Wallis’ analyses. Rank-biserial correlation (*r*_*rb*_) was calculated for the key peritonitis + SJWO *versus* peritonitis + saline pairwise comparisons. Ninety-five per cent confidence intervals (95%CI) for effect sizes were estimated using 5,000 stratified non-parametric bootstrap resamples within experimental groups. A two-sided *p* < 0.05 was considered statistically significant.

## Results

The biochemical parameters, peritoneal thickness measurements, and histopathological scores of all groups are summarized in Tables 3 and 4.

**Table 3:** Biochemical parameters, body-weight change, and peritoneal thickness across the study groups<tfn href="tfn01">*</tfn>.

Variable	Control (n = 6)	SJWO (n = 6)	Olive oil (n = 6)	Peritonitis (n = 6)	Peritonitis + saline (n = 6)	Peritonitis + SJWO (n = 6)	Peritonitis + olive oil (n = 6)	*p* -value effect size (95%CI)
Pre-procedure weight (g)	245.0 ± 5.8 Ψ,¥ 243.0 (240.0–256.0)	262.3 ± 4.8 261.0 (258.0–270.0)	248.7 ± 10.6 252.0 (230.0–258.0)	272.7 ± 13.8 α 268.0 (262.0–300.0)	246.3 ± 12.2 242.0 (238.0–270.0)	275.0 ± 19.8 280.0 (244.0–294.0)	262.0 ± 11.5 258.0 (252.0–280.0)	0.001^a^ ε^2^ = 0.498 (0.330–0.807)
Post-procedure weight (g)	244.3 ± 7.5 244.0 (234.0–256.0)	255.7 ± 5.4 254.0 (252.0–266.0)	246.3 ± 9.8 247.0 (230.0–256.0)	248.3 ± 20.1 245.0 (222.0–284.0)	228.7 ± 22.1 235.0 (200.0–256.0)	261.3 ± 21.1 270.0 (232.0–284.0)	246.0 ± 9.0 245.0 (234.0–258.0)	0.107^a^ ε^2^ = 0.127 (0.016–0.580)
Weight change (%)	-0.2 ± 3.9 Ψ -1.6 (-3.9–5.8)	-2.5 ± 2.3 -2.3 (-6.7–0.0)	-0.9 ± 1.1 Ψ -0.8 (-3.1–0.0)	-9.0 ± 4.5 -8.6 (-17.2 to -4.6)	-7.3 ± 6.5 -4.2 (-16.0 to -1.7)	-5.0 ± 2.6 -5.3 (-8.5 to -1.5)	-6.0 ± 3.5 -6.0 (-11.4 to -0.8)	0.002^a^ ε^2^ = 0.424 (0.308–0.692)
CRP (mg/L)	5.6 ± 0.7 Ψ,¤,ϕ,φ 5.7 (4.4–6.3)	8.3 ± 0.8 ¥ 8.5 (7.2–9.1)	7.7 ± 0.3 ¥ 7.7 (7.4–8.2)	8.0 ± 1.3 7.8 (6.1–9.6)	7.0 ± 0.8 6.9 (5.7–7.9)	5.6 ± 1.0 5.5 (4.4–7.3)	7.2 ± 0.7 7.3 (6.1–7.9)	< 0.001^b^ ω^2^ = 0.557 (0.441–0.785)
Procalcitonin (pg/mL)	248.0 ± 32.5 φ 256.3 (194.2–283.8)	320.3 ± 34.5 322.0 (283.0–378.0)	339.8 ± 34.9 ¥ 342.5 (299.0–398.0)	293.2 ± 68.2 289.2 (205.0–394.7)	275.3 ± 31.4 271.5 (242.0–334.0)	253.8 ± 28.5 248.2 (215.8–289.8)	294.2 ± 30.2 293.9 (247.7–332.3)	0.002^b^ ω^2^ = 0.323 (0.223–0.631)
IL-1 (pg/mL)	27.1 ± 10.3 φ 26.2 (16.9–44.9)	38.7 ± 3.0 38.5 (35.0–43.0)	43.8 ± 2.7 Ψ 43.0 (42.0–49.0)	29.7 ± 3.9 28.2 (26.3–34.8)	35.7 ± 5.8 35.5 (28.0–44.0)	34.4 ± 4.9 34.6 (26.7–41.2)	35.1 ± 5.9 35.3 (28.6–42.9)	0.003^a^ ε^2^ = 0.387 (0.249–0.724)
IL-6 (pg/mL)	25.3 ± 3.6 Ψ,φ 24.9 (21.3–31.4)	37.2 ± 7.4 38.0 (24.0–46.0)	40.7 ± 8.7 41.5 (27.0–50.0)	35.9 ± 4.2 35.1 (30.0–41.3)	34.3 ± 10.5 34.0 (20.0–52.0)	31.4 ± 4.2 32.1 (24.4–36.5)	26.6 ± 10.2 28.4 (7.4–35.2)	0.010^a^ ε^2^ = 0.299 (0.149–0.672)
TNF-α (pg/mL)	76.7 ± 17.6 ¤ 75.2 (50.0–100.9)	101.3 ± 10.0 104.5 (82.0–108.0)	100.3 ± 9.0 100.5 (84.0–110.0)	97.8 ± 29.8 86.4 (76.8–157.3)	93.3 ± 9.0 95.0 (78.0–102.0)	102.8 ± 13.8 107.1 (83.7–118.0)	109.6 ± 11.1 111.1 (90.8–119.8)	0.015^a^ ε^2^ = 0.280 (0.123–0.663)
Visceral peritoneal thickness (μm)	77.9 ± 43.1 74.1 (27.0–151.6)	108.0 ± 156.3 α 39.5 (20.0–420.0)	26.7 ± 10.0 α,¥ 24.0 (17.0–44.0)	309.4 ± 244.0 337.1 (21.4–609.6)	1151.3 ± 843.6 941.0 (438.0–2,707.0)	971.1 ± 1,040.9 438.6 (163.4–2,498.9)	335.4 ± 241.2 309.4 (86.1–657.7)	< 0.001^a^ ε^2^ = 0.601 (0.532–0.785)
Parietal peritoneal thickness (μm)	85.5 ± 49.6 α 77.0 (41.3–174.5)	356.0 ± 237.2 307.5 (117.0–740.0)	180.8 ± 96.3 170.5 (78.0–330.0)	227.3 ± 215.9 210.0 (29.0–620.0)	604.3 ± 617.4 423.5 (143.0–1,764.0)	205.4 ± 259.1 86.0 (65.3–725.0)	232.4 ± 132.7 210.6 (105.4–385.8)	0.049^a^ ε^2^ = 0.189 (0.076–0.566)

* Data are presented as mean ± standard deviation and median (minimum–maximum). Pairwise comparisons used the Mann–Whitney’s U test with Bonferroni correction or the Bonferroni post-hoc test. Omnibus effect sizes are reported as ε^2^ for Kruskal–Wallis’ analyses and ω^2^ for one-way analysis of variance, with 95% confidence intervals (95%CI) obtained from 5,000 stratified non-parametric bootstrap resamples within experimental groups;

a Kruskal–Wallis’ test;

b one-way analysis of variance; ϕ: *p* < 0.05 considered statistically significant versus SJWO; φ: *p* < 0.05 considered statistically significant *versus* olive oil; Ψ: *p* < 0.05 considered statistically significant *versus* peritonitis; α: *p* < 0.05 considered statistically significant *versus* peritonitis + saline; ¥: *p* < 0.05 considered statistically significant *versus* peritonitis + SJWO; ¤: *p* < 0.05 considered statistically significant *versus* peritonitis + olive oil; CRP: C-reactive protein; IL: interleukin; TNF: tumor necrosis factor alpha;

SJWO: St. John’s Wort oil.

Source: Elaborated by the authors.

**Table 4:** Histopathological scores (inflammation, fibrosis, and vascular proliferation) of the visceral and parietal peritoneum<tfn>*</tfn>.

Variable	Control (n = 6)	SJWO (n = 6)	Olive oil (n = 6)	Peritonitis (n = 6)	Peritonitis + saline (n = 6)	Peritonitis + SJWO (n = 6)	Peritonitis + olive oil (n = 6)	*p* -value effect size (95% CI)
Visceral inflammation	0.5 (0–1) α,¥,¤	1.0 (0–2)	1.0 (0–1) α,¤	2.0 (1–3)	3.0 (3–3)	3.0 (2–3)	3.0 (3–3)	< 0.001 ε^2^ = 0.792 (0.754–0.895)
Visceral fibrosis	0.0 (0–1) α	0.0 (0–2) α	0.0 (0–1) α	2.0 (1–2)	3.0 (2–3)	2.0 (1–2)	2.0 (1–2)	< 0.001 ε^2^ = 0.679 (0.586–0.893)
Visceral vascular proliferation	0.0 (0–1) α,¥,¤	0.0 (0–2) α,¤	0.0 (0–1) α,¥,¤	2.0 (1–3)	3.0 (3–3)	3.0 (2–3)	3.0 (3–3)	< 0.001 ε^2^ = 0.807 (0.768–0.919)
Parietal inflammation	0.0 (0–1) Ψ,α	1.0 (0–2)	1.0 (1–2)	3.0 (0–3)	3.0 (1–3)	1.0 (0–1)	1.0 (1–3)	0.001 ε^2^ = 0.504 (0.386–0.798)
Parietal fibrosis	0.0 (0–0) α	0.0 (0–1)	1.0 (0–1)	3.0 (0–3)	2.0 (0–3) ¥	0.0 (0–0)	1.0 (1–3)	0.006 ε^2^ = 0.348 (0.181–0.741)
Parietal vascular proliferation	0.0 (0–0) α	0.0 (0–2)	1.0 (0–2)	3.0 (0–3)	3.0 (0–3) ¥	0.0 (0–0)	0.5 (0–3)	0.006 ε^2^ = 0.349 (0.184–0.724)

* Data are presented as median (minimum–maximum).

Scores range from 0 to 3 (0 = normal, 1 = mild, 2 = moderate, 3 = severe). Between-group comparisons used the Kruskal–Wallis’ test; pairwise comparisons used the Mann–Whitney’s U test with Bonferroni correction. Omnibus effect sizes are reported as ε^2^ with 95% confidence intervals (95%CI) obtained from 5,000 stratified non-parametric bootstrap resamples within experimental groups; ϕ: *p* < 0.05 considered statistically significant *versus* SJWO; φ: *p* < 0.05 considered statistically significant *versus* olive oil; Ψ: *p* < 0.05 considered statistically significant *versus* peritonitis; α: *p* < 0.05 considered statistically significant *versus* peritonitis + saline; ¥: *p* < 0.05 considered statistically significant *versus* peritonitis + SJWO; ¤: *p* < 0.05 considered statistically significant *versus* peritonitis + olive oil; SJWO: St. John’s Wort oil. Source: Elaborated by the authors.

### Body weight change

The percentage change in body weight differed significantly among the seven groups (*p* = 0.002). The greatest weight loss was observed in the peritonitis group; in pairwise comparisons, the differences between the control–peritonitis and olive oil–peritonitis groups were statistically significant (*p* < 0.05). Post-treatment absolute body weights did not differ significantly among groups (*p* = 0.107). The between-group effect size was ε^2^ = 0.424 (95%CI 0.308–0.692) for percentage weight change and ε^2^ = 0.127 (95%CI 0.016–0.580) for post-treatment absolute body weight.

### Acute-phase reactants

CRP (*p* < 0.001) and procalcitonin (*p* = 0.002) levels differed significantly among groups. The CRP level in the control group was significantly lower than in the peritonitis, peritonitis + olive oil, SJWO, and olive oil groups; the SJWO and olive oil groups had significantly higher levels than the peritonitis + SJWO group. For procalcitonin, only the control–olive oil and olive oil–peritonitis + SJWO comparisons were significant. The omnibus effect sizes were ω^2^ = 0.557 (95%CI 0.441–0.785) for CRP and ω^2^ = 0.323 (95%CI 0.223–0.631) for procalcitonin.

### Pro-inflammatory cytokines

IL-1 (*p* = 0.003), IL-6 (*p* = 0.010), and TNF-α (*p* = 0.015) levels differed significantly among groups. In pairwise comparisons, the differences were significant for IL-1 between control–olive oil and olive oil–peritonitis; for IL-6 between control–peritonitis and control–olive oil; and for TNF-α only between control–peritonitis + olive oil (all *p* < 0.05). Notably, cytokine levels in the SJWO and olive oil groups were elevated compared with controls. The omnibus effect sizes were ε^2^ = 0.387 (95%CI 0.249–0.724) for IL-1, ε^2^ = 0.299 (95%CI 0.149–0.672) for IL-6, and ε^2^ = 0.280 (95%CI 0.123–0.663) wfor TNF-α.

### Peritoneal thickness

Visceral peritoneal thickness differed significantly among groups (*p* < 0.001); pairwise comparisons revealed significant differences for SJWO–peritonitis + saline, olive oil–peritonitis + saline, and olive oil–peritonitis + SJWO. Parietal peritoneal thickness showed significance of lower magnitude (*p* = 0.049), driven solely by the control–peritonitis + saline comparison. In both anatomical sites, the thickest peritoneum was observed in the peritonitis + saline group; among the peritonitis-induced groups, the thinnest parietal peritoneum was measured in the peritonitis + SJWO group. The omnibus effect size was ε^2^ = 0.601 (95%CI 0.532–0.785) for visceral thickness and ε^2^ = 0.189 (95%CI 0.076–0.566) for parietal thickness.

### Visceral peritoneal histopathology

Inflammation, fibrosis, and vascular proliferation scores all differed significantly among groups (*p* < 0.001). For inflammation, the control group was significantly lower than the peritonitis + saline, peritonitis + SJWO, and peritonitis + olive oil groups. The fibrosis score was the highest in the peritonitis + saline group, with statistically significant differences compared with the control, SJWO, and olive oil groups. For visceral vascular proliferation, the control and olive oil groups differed significantly from all three peritonitis-treatment groups, whereas the SJWO group differed significantly from the peritonitis + saline and peritonitis + olive oil groups (Fig. 2). The omnibus ε2 values were 0.792 (95%CI 0.754–0.895) for inflammation, 0.679 (95%CI 0.586–0.893) for fibrosis, and 0.807 (95%CI 0.768–0.919) for vascular proliferation.

**Figure 2:**
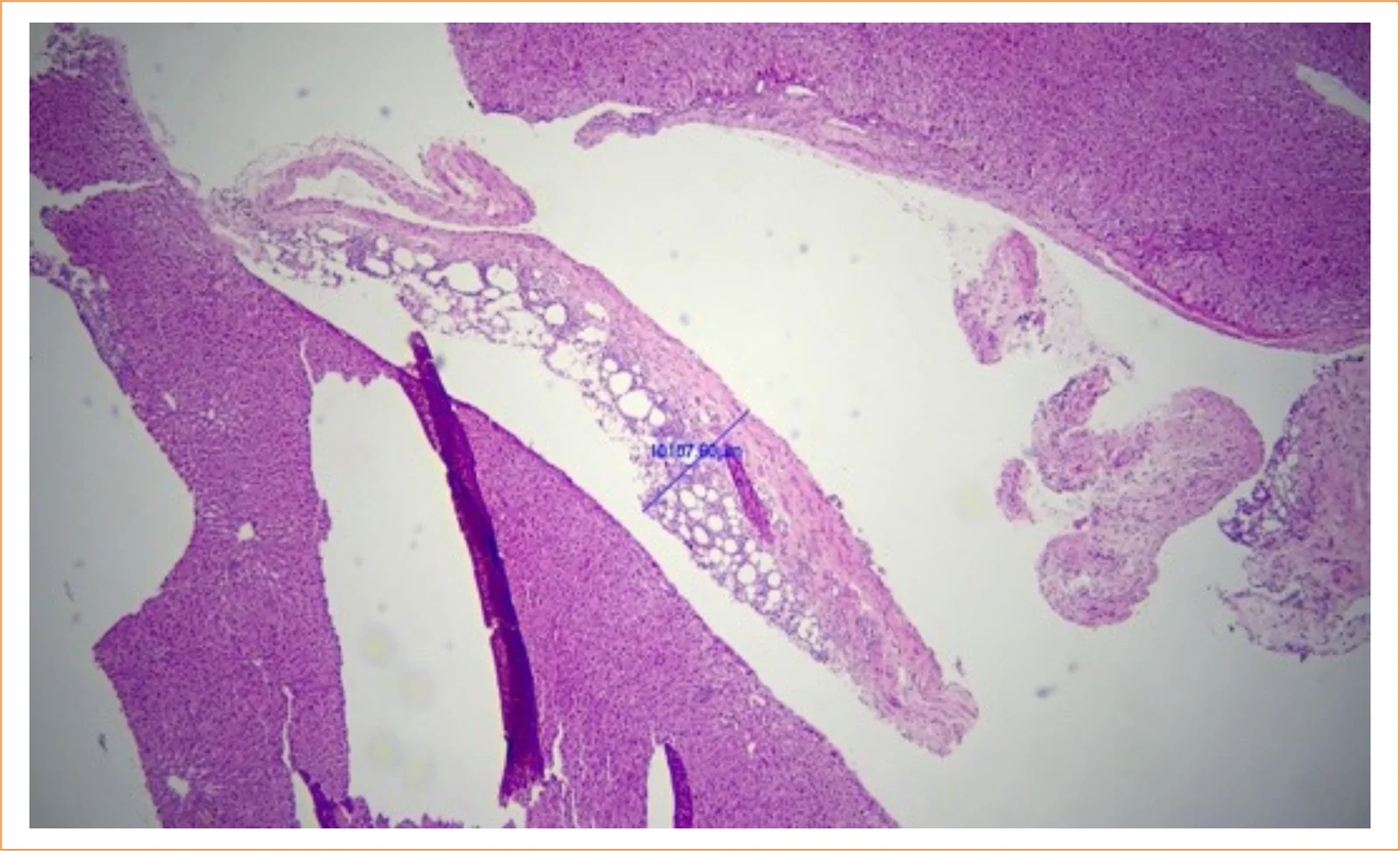
Photomicrograph of the visceral peritoneum (hepatic capsule) in the peritonitis + St. John’s Wort oil group showing marked inflammation and fibrosis. Hematoxylin-eosin staining; original magnification, ×400.

Source: Elaborated by the authors.

### Parietal peritoneal histopathology

Inflammation (*p* = 0.001), fibrosis (*p* = 0.006), and vascular proliferation (*p* = 0.006) scores differed significantly among groups. For inflammation, the control–peritonitis and control–peritonitis + saline comparisons were significant (Fig. 3). For both fibrosis and vascular proliferation, scores were lower in the peritonitis + SJWO group than in the peritonitis + saline group. The magnitude of each pairwise difference was large (*r*_*rb*_ = -0.833; 95%CI -1.000 to -0.500). The omnibus ε^2^ values were 0.504 (95%CI 0.386–0.798) for inflammation, 0.348 (95%CI 0.181–0.741) for fibrosis, and 0.349 (95%CI 0.184–0.724) for vascular proliferation. These findings demonstrated a local histopathological difference at day 5 but do not by themselves establish prevention of adhesion formation.

**Figure 3:**
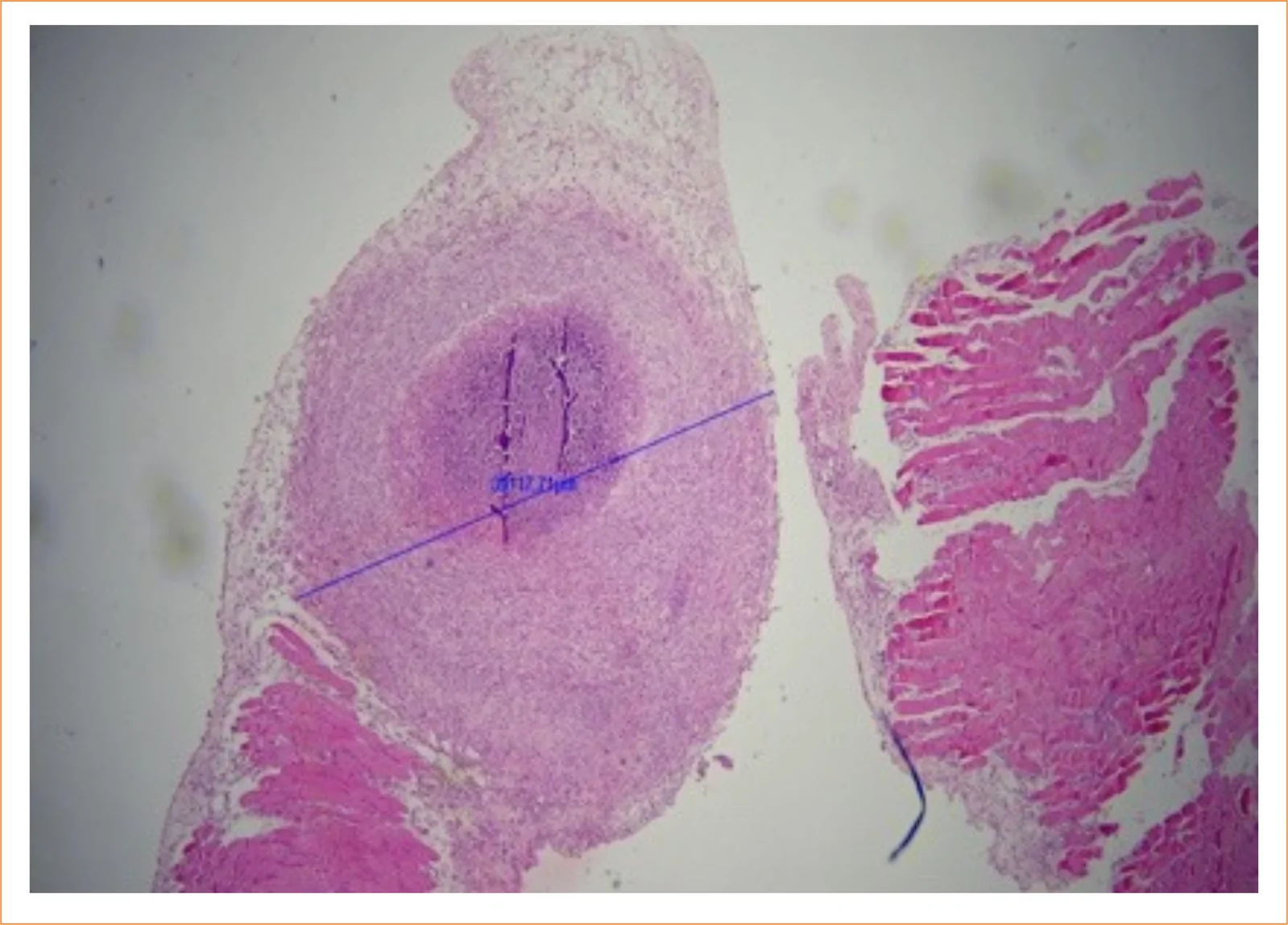
Photomicrograph of the parietal peritoneum in the peritonitis group showing marked inflammation and fibrosis. Hematoxylin-eosin staining; original magnification, ×400.

Source: Elaborated by the authors.

## Discussion

This study examined intraperitoneal *H. perforatum* oil (SJWO) in a feces-induced experimental peritonitis model. The findings were anatomically and biologically discordant: SJWO did not produce a consistent reduction in systemic inflammatory markers or visceral fibrosis, whereas parietal fibrosis and vascular proliferation scores were lower than those observed after saline treatment. Accordingly, the local parietal findings should be interpreted as exploratory histopathological signals rather than evidence of a general anti-inflammatory or anti-adhesive effect.

### Systemic inflammatory response

Prior experimental studies indicate that the inflammatory response in peritonitis is highly dependent on the causal model, intervention, timing, and compartment sampled. Hyaluronic acid, mesalazine, adiponectin, selected Allium sativum extracts, N-salicyloyl tryptamine, simvastatin, probiotic pre-colonization, and antibiotic lavage have each modified inflammatory or survival outcomes in distinct models, but their targets and readouts were not uniform^8-15^. Likewise, clinical studies supporting procalcitonin or CRP as diagnostic biomarkers do not establish that an intraperitoneal intervention will reduce these markers^16-18^. These differences limit direct efficacy comparisons and provide a framework for interpreting the absence of systemic suppression with SJWO in the present study.

In our experiments, SJWO did not consistently suppress systemic inflammation. Some cytokines and acute-phase reactants were also higher in the non-peritonitis SJWO or olive-oil groups than in control ones, suggesting that exposure of the peritoneal cavity to an oil-based preparation may itself influence immune signaling. This contrasts with the reductions in IL-1 and IL-6 reported after oral olive oil in an experimental colitis model^19^ and with the antioxidant and tissue-protective effects of intraperitoneal *Hypericum* extract reported in TNBS-induced colitis^20^. The discrepancy may reflect differences in the inflammatory model, formulation, composition, dose, and tissue compartment rather than route alone. A recent review similarly emphasized that *H. perforatum* preparations may influence cytokine production, fibroblast behavior, collagen deposition, and angiogenesis, with effects depending on their phytochemical composition and experimental context^21^. Because the commercial preparation used here was not quantitatively standardized for hypericin or hyperforin, a constituent-specific mechanism cannot be inferred.

### Peritoneal thickness and local histopathology

Local peritoneal remodeling involves linked but non-identical processes, including inflammation, mesothelial injury, and mesothelial-to-mesenchymal transition, extracellular-matrix deposition, fibroblast activation, angiogenesis, and fibrinolytic imbalance. Recent reviews emphasize that these pathways vary over time and that histological fibrosis or neovascularization at a single early time point cannot be treated as a surrogate for mature adhesion formation^22,23^. Experimental studies of antithrombin III, stem-cell-based approaches, valproic acid, and anti-exudative agents nevertheless support the biological relevance of fibrosis and vascular responses as local exploratory endpoints^24-27^.

In our study, the higher peritoneal thickness and histopathological scores in peritonitis-exposed groups support successful induction of local injury. However, SJWO did not reduce visceral fibrosis, and the lower fibrosis and vascular proliferation scores were confined to the parietal peritoneum relative to peritonitis + saline. This anatomical discordance argues against a uniform antifibrotic effect and could reflect heterogeneous intraperitoneal distribution, surface contact, or tissue-specific repair; none of these mechanisms was directly measured. Hizli et al.^7^ used 2 mL/rat of intraperitoneal *H. perforatum* in a 14-day postoperative uterine-horn adhesion model and found no significant reduction in adhesions, inflammation, or fibrosis. By contrast, our study used a 1-mL dose in an infectious peritonitis model and evaluated early histopathology on day 5 without macroscopic adhesion scoring. Differences in model, dose, follow-up, and endpoints therefore preclude direct comparison. The present parietal findings may justify hypothesis-driven evaluation in a dedicated adhesion model, but they cannot be interpreted as evidence that SJWO prevents adhesions.

### Study limitations

Our study has several limitations. The sample size was small (n = 6 per group), and no *a priori* power calculation was performed; consequently, estimates were imprecise, as reflected by several wide confidence intervals, and both false-negative and unstable positive findings were possible. The analysis was restricted to plasma inflammatory markers and routine hematoxylin-eosin histopathology; anti-inflammatory cytokines, oxidative-stress markers, tissue cytokines, microbiological burden, and pathway-specific molecular or immunohistochemical markers were not assessed.

Although the fecal suspension was standardized procedurally, viable bacterial counts and microbial composition were not quantified, and the use of autologous feces may have introduced biological heterogeneity. The commercial SJWO preparation lacked a recorded lot number and quantitative phytochemical standardization, and hypericin/hyperforin concentrations were unavailable. Only one dose and one early endpoint (day 5) were studied.

Adhesions were not scored macroscopically, and the short follow-up was insufficient to evaluate mature adhesion formation. Therefore, the fibrosis and vascular proliferation findings could not be extrapolated to adhesion prevention. Leukocyte data could not be analyzed because sufficient blood volume was not obtained. Finally, baseline body weights differed among groups; percentage weight change was used as the within-animal weight outcome, but residual imbalance remains possible.

## Conclusion

In this feces-induced experimental peritonitis model, intraperitoneal SJWO did not produce a consistent systemic anti-inflammatory effect. Although parietal peritoneal fibrosis and vascular proliferation scores were lower with SJWO than with saline, visceral fibrosis was not reduced, and the findings were limited to a single early histological assessment. Because adhesion formation was not directly evaluated, no conclusion could be drawn regarding adhesion prevention. Further studies should use chemically standardized preparations, predefined sample-size calculations, microbiologically standardized inocula, multiple doses and time points, and dedicated macroscopic and molecular adhesion endpoints before intraperitoneal application must be considered.

## Data Availability

The datasets generated and/or analyzed during the current study are available from the corresponding author on reasonable request.

## References

[1] Kirkpatrick AW, Coccolini F, Ansaloni L, Roberts DJ, Tolonen M, McKee JL, Leppaniemi A, Faris P, Doig CJ, Catena F, Fabian T, Jenne CN, Chiara O, Kubes P, Manns B, Kluger Y, Fraga GP, Pereira BM, Diaz JJ, Sugrue M, Moore EE, Ren J, Ball CG, Coimbra R, Balogh ZJ, Abu-Zidan FM, Dixon E, Biffl W, MacLean A, Ball I, Drover J, McBeth PB, Posadas-Calleja JG, Parry NG, Di Saverio S, Ordoñez CA, Xiao J, Sartelli M (2018). Closed Or Open after Source Control Laparotomy for Severe Complicated Intra-Abdominal Sepsis (the COOL trial): study protocol for a randomized controlled trial. World J Emerg Surg.

[2] Lodato RF, Khan AR, Zembowicz MJ, Weisbrodt NW, Pressley TA, Li YF, Lodato JA, Zembowicz A, Moody FG (1999). Roles of IL-1 and TNF in the decreased ileal muscle contractility induced by lipopolysaccharide. Am J Physiol.

[3] Koç TR, Tarhan ÖR, Sarıçık B (2018). Effects of peritoneal lavage and dry cleaning on bacterial translocation in a model of peritonitis developed using cecal ligation and puncture. Ulus Travma Acil Cerrahi Derg.

[4] Fernández LG, Matthews MR, Seal L (2020). Intraabdominal lavage of hypochlorous acid: a new paradigm for the septic and open abdomen. Wounds.

[5] Eğilmez OK, Kökten N, Ekici AİD, Kalcıoğlu MT, Yeşilada E, Tekin M (2015). The effect of Hypericum perforatum L. (St. John’s Wort) on prevention of myringosclerosis after myringotomy in a rat model. Int J Pediatr Otorhinolaryngol.

[6] Yücel A, Kan Y, Yeşilada E, Akın O (2017). Effect of St. John’s wort (Hypericum perforatum) oily extract for the care and treatment of pressure sores: a case report. J Ethnopharmacol.

[7] Hızlı D, Hızlı F, Köşüş A, Yılmaz S, Köşüş N, Haltaş H, Dede H, Kafalı H (2014). Effect of Hypericum perforatum on intraperitoneal adhesion formation in rats. Arch Med Sci.

[8] Lee JH, Liu A, Park JH, Kato H, Hao Q, Zhang X, Zhou L, Lee JW (2020). Therapeutic effects of hyaluronic acid in peritonitis-induced sepsis in mice. Shock.

[9] Şener A, Şahbaz A, Şener LT, Tekkeşin MS, Kaya B (2018). Effect of intra-abdominally administered mesalazine (5-aminosalicylic acid) in experimental peritonitis. North Clin Istanb.

[10] Salman B, Yılmaz TU, Tezcaner T, Demir EO, Paşaoğlu OT (2014). Exogenous recombinant adiponectin improves survival in experimental abdominal sepsis. Balkan Med J.

[11] Redondo-Calvo FJ, Bejarano-Ramírez N, Baladrón V, Montenegro O, Gómez LA, Velasco R, Villasanti N, Illescas S, Franco-Sereno MT, Gracia I, Rodríguez JF, Muñoz-Rodríguez JR, Pérez-Ortiz JM (2022). Black garlic and thiosulfinate-enriched extracts as adjuvants to ceftriaxone treatment in a rat peritonitis model of sepsis. Biomedicines.

[12] Sousa-Neto BP, Cunha FVM, Nunes DB, Gomes BS, Amorim LV, Lopes EM, Gutierrez SJC, Almeida FRC, Arcanjo DDR, Souza MFV, Oliveira FA (2022). Anti-inflammatory and antioxidant effects of the indole-derived N-salicyloyltryptamine on peritonitis and joint disability induced by carrageenan in rodents. Evid Based Complement Alternat Med.

[13] Altunal C, Agalar F, Agalar C, Daphan Ç, Saygun O, Aydinuraz K, Sahiner T, Atasoy P, Caglayan O, Dom S (2015). The effect of simvastatin on pulmonary damage in experimental peritonitis in rats. Indian J Surg.

[14] Gutiérrez-Falcón AI, Ramos-Nuez AM, Monteros y Zayas AE, Castillo DFP, García-Laorden MI, Chamizo-López FJ, Valcárcel FR, Campelo FA, Benítez AB, Salgueiro PN, Cabrera CD, Rivero-Vera JC, González-Martín JM, Caballero JM, Frías-Beneyto R, Villar J, Martín-Barrasa JL (2021). Probiotic properties of Alcaligenes faecalis isolated from Argyrosomus regius in experimental peritonitis (rat model). Probiotics Antimicrob Proteins.

[15] Perdue PW, Kazarian KK, Nevola J, Law WR, Williams T (1994). The use of local and systemic antibiotics in rat fecal peritonitis. J Surg Res.

[16] Yang SK, Xiao L, Zhang H, Xu XX, Song PA, Liu FY, Sun L (2014). Significance of serum procalcitonin as biomarker for detection of bacterial peritonitis: a systematic review and meta-analysis. BMC Infect Dis.

[17] Abdel-Razik A, Mousa N, Elhammady D, Elhelaly R, Elzehery R, Elbaz S, Eissa M, El-Wakeel N, Eldars W (2016). Ascitic fluid calprotectin and serum procalcitonin as accurate diagnostic markers for spontaneous bacterial peritonitis. Gut Liver.

[18] Yücel T, Gönüllü D, Güçlü S, Şit M, Adaleti R, Tetikkurt S, Özcan A, Köksoy FN (2008). The therapeutic effect of normobaric oxygen in experimental peritonitis and the efficiency of rectal fever, WBC, CRP and procalcitonin in monitoring response of the therapy [in Turkish]. Ulus Travma Acil Cerrahi Derg.

[19] Cariello M, Contursi A, Gadaleta RM, Piccinin E, De Santis S, Piglionica M, Spaziante AF, Sabbà C, Villani G, Moschetta A (2020). Extra-virgin olive oil from Apulian cultivars and intestinal inflammation. Nutrients.

[20] Dost T, Ozkayran H, Gokalp F, Yenisey C, Birincioglu M (2009). The effect of Hypericum perforatum (St. John’s Wort) on experimental colitis in rat. Dig Dis Sci.

[21] Far BF, Gouranmohit G, Naimi-Jamal MR, Neysani E, El-Nashar HAS, El-Shazly M, Khoshnevisan K (2024). The potential role of Hypericum perforatum in wound healing: a literature review on the phytochemicals, pharmacological approaches, and mechanistic perspectives. Phytother Res.

[22] Stratakis K, Kalogiannis E, Thanopoulos A, Grigoriadis G, Roditis S, Kaliatsi EG, Nikiteas N, Kontzoglou K, Perrea D (2023). Mechanisms and therapeutic approaches for peritoneal adhesions: a comprehensive review. Chirurgia (Bucur).

[23] Kang Y, Liu Y, Fu P, Ma L (2024). Peritoneal fibrosis: from pathophysiological mechanism to medicine. Front Physiol.

[24] Akman S, Koyun M, Gelen T, Coskun M (2008). Comparison of intraperitoneal antithrombin III and heparin in experimental peritonitis. Pediatr Nephrol.

[25] Alatab S, Najafi I, Atlasi R, Pourmand G, Tabatabaei-Malazy O, Ahmadbeigi N (2018). A systematic review of preclinical studies on therapeutic potential of stem cells or stem cells products in peritoneal fibrosis. Minerva Urol Nefrol.

[26] Costalonga EC, Freitas LJ, Aragone DDSP, Silva FMO, Noronha IL (2017). Anti-fibrotic effects of valproic acid in experimental peritoneal fibrosis. PLoS One.

[27] Kudryashov NV, Ivanova EA, Kalinina TS, Shimshirt AA, Kurshin AA, Zhmurenko LA, Voronina TA (2020). Antiexudative effects of finasteride and a new pyrazolo[c]pyridine derivative GIZh-72 in acetic acid-induced experimental peritonitis. Bull Exp Biol Med.

